# Functional Pituitary Networks in Vertebrates

**DOI:** 10.3389/fendo.2020.619352

**Published:** 2021-01-27

**Authors:** Yorgui Santiago-Andres, Matan Golan, Tatiana Fiordelisio

**Affiliations:** ^1^ Laboratorio de Neuroendocrinología Comparada, Departamento de Ecología y Recursos Naturales, Biología, Facultad de Ciencias, Universidad Nacional Autónoma de México, Ciudad Universitaria, Ciudad de México, Mexico; ^2^ Department of Poultry and Aquaculture, Institute of Animal Sciences, Agricultural Research Organization, Rishon Lezion, Israel

**Keywords:** pituitary, networks, plasticity, vertebrates, evolution

## Abstract

The pituitary is a master endocrine gland that developed early in vertebrate evolution and therefore exists in all modern vertebrate classes. The last decade has transformed our view of this key organ. Traditionally, the pituitary has been viewed as a randomly organized collection of cells that respond to hypothalamic stimuli by secreting their content. However, recent studies have established that pituitary cells are organized in tightly wired large-scale networks that communicate with each other in both homo and heterotypic manners, allowing the gland to quickly adapt to changing physiological demands. These networks functionally decode and integrate the hypothalamic and systemic stimuli and serve to optimize the pituitary output into the generation of physiologically meaningful hormone pulses. The development of 3D imaging methods and transgenic models have allowed us to expand the research of functional pituitary networks into several vertebrate classes. Here we review the establishment of pituitary cell networks throughout vertebrate evolution and highlight the main perspectives and future directions needed to decipher the way by which pituitary networks serve to generate hormone pulses in vertebrates.

## Introduction

Biological variation is a defining characteristic in the process of species evolution and adaptation. Variation in morphology, physiology, or behavior within species is a prerequisite for evolutionary processes, like natural selection, to occur. In an ever-changing natural world, the organisms able to respond more efficiently to those changes are better fitted to their environment and could be selected (1). In this context, highly plastic systems are also adaptive since the information from both external and internal environments is better integrated. An example of such a highly plastic system is the pituitary gland, which secretes several key hormones that maintain homeostasis in all vertebrates (2).

The pituitary gland is embryonically derived from Rathke’s pouch and is therefore considered to be of ectodermal origin (3, 4), although at least in fish, a small percentage of pituitary endocrine cells have endodermal origins (5). The pituitary of all vertebrates consists of three anatomically and developmentally distinct structures called the neurohypophysis or posterior pituitary, the adenohypophysis, or anterior lobe and the intermediate lobe, all of which function in close interdependence with the hypothalamus. The neurohypophysis comprises the neural lobe (mainly oxytocin- and vasopressin-secreting neuroendocrine terminals in mammals and their homologous isotocin and vasotocin in non-mammalian vertebrates), the median eminence (the functional link between the hypothalamus and the anterior lobe), and the infundibulum. The intermediate lobe is mainly composed of melanotrophs that regulate skin pigmentation. The anterior lobe of the pituitary, which is the main focus of this review, is populated by five types of secretory cells: somatotrophs, gonadotrophs, lactotrophs, thyrotrophs, and corticotrophs, that may be identified by their hormone composition and their response to hypothalamic neurohormones. In mammals, these cell types follow a similar pattern of stimulus-secretion coupling that regulates the neuroendocrine axes and allows the preservation of homeostasis (6).

In all vertebrates, the main driver of pituitary secretion is the hypothalamic input. Factors released from hypothalamic neuron terminals into the pituitary portal system bind to their cognate receptors on target pituitary cells and elicit a signaling cascade that induces release (or inhibition) of hormone secretion from the pituitary (7) into the vascular system in a pulsatile manner, the pattern of which is critical in regulating the activity of the downstream target organs (2). Yet the individual cellular dynamics *in vitro* do not recapitulate the whole gland pulse generation needed for physiological actions. For example, *in vivo*, GH-secreting cells produce massive GH pulses (up to 1,000-fold rise) in response to physiological cues  (8, 9), whereas these pulses are significantly weaker *in vitro* (10). The same dependence of hormone pulse generation on the tissue context has been observed in gonadotrophs (11–14), corticotrophs (15–17), and lactotrophs (18, 19). Thus, in order to generate this fine-tuned and coordinated secretion, a large-scale communication system must exist within the anterior pituitary that is critical for the ability to generate hormone surges.

In recent years, the use of high resolution imaging in combination with large-scale cell activity recordings has revealed the homotypic and heterotypic network arrangement of the mammalian pituitary cells (i.e., intercellular connectivity between the same secretory cell type or between different cell types, respectively). Research in this field has pointed out the relevance of the network organization in coordinating the synchronized and rhythmic secretion of hormones (16, 20). Intriguingly, the existence of cell networks has been identified in all major groups of vertebrates. As an example, in fish, which represent the most basal group of vertebrates, when the gonadotroph network connectivity is disrupted, the hormone secretion is compromised, as well as its biological significance (21, 22). It can be concluded from these facts that a) pulse generation is achieved thanks to cell networks, b) the importance of the pulse mode of secretion is revealed by studies in which its production is limited, and is followed by physiological perturbations in the target organs of regulation, and c) cell network formation, which is essential for the organization of these pulses, is conserved throughout vertebrate evolution (2).

Despite the clear importance of pituitary homotypic and heterotypic networks in the coordination of hormone pulsatility, research into this subject has been limited. Here, we review the current knowledge and methodological approaches to assess the function of pituitary networks throughout vertebrate evolution. The mechanisms involved in pulse generation, as an adaptive and plastic process that promotes the rhythmic secretion of hormones in vertebrates, will be discussed from a cell network perspective. We then explore the evolution of these networks in different groups of vertebrates, from fish to mammals and discuss the importance of the cellular networks in the phenotypic plasticity. We conclude that a comparative approach to the study of pituitary networks is fundamental to the comprehension of the system both under normal and pathological conditions. It is important to clarify that factors such as the well-established hypothalamic drive, the peripheral tissues and the environment are fundamental in the generation of hormone pulses and should be integrated in order to understand the system as a whole. However, the pituitary gland has its own local and plastic modes of autonomic regulation and its interaction with the hypothalamus is a complex two-ways regulation.

## Hypothalamic Control of the Pituitary Gland in Vertebrates

**Figure 1:**
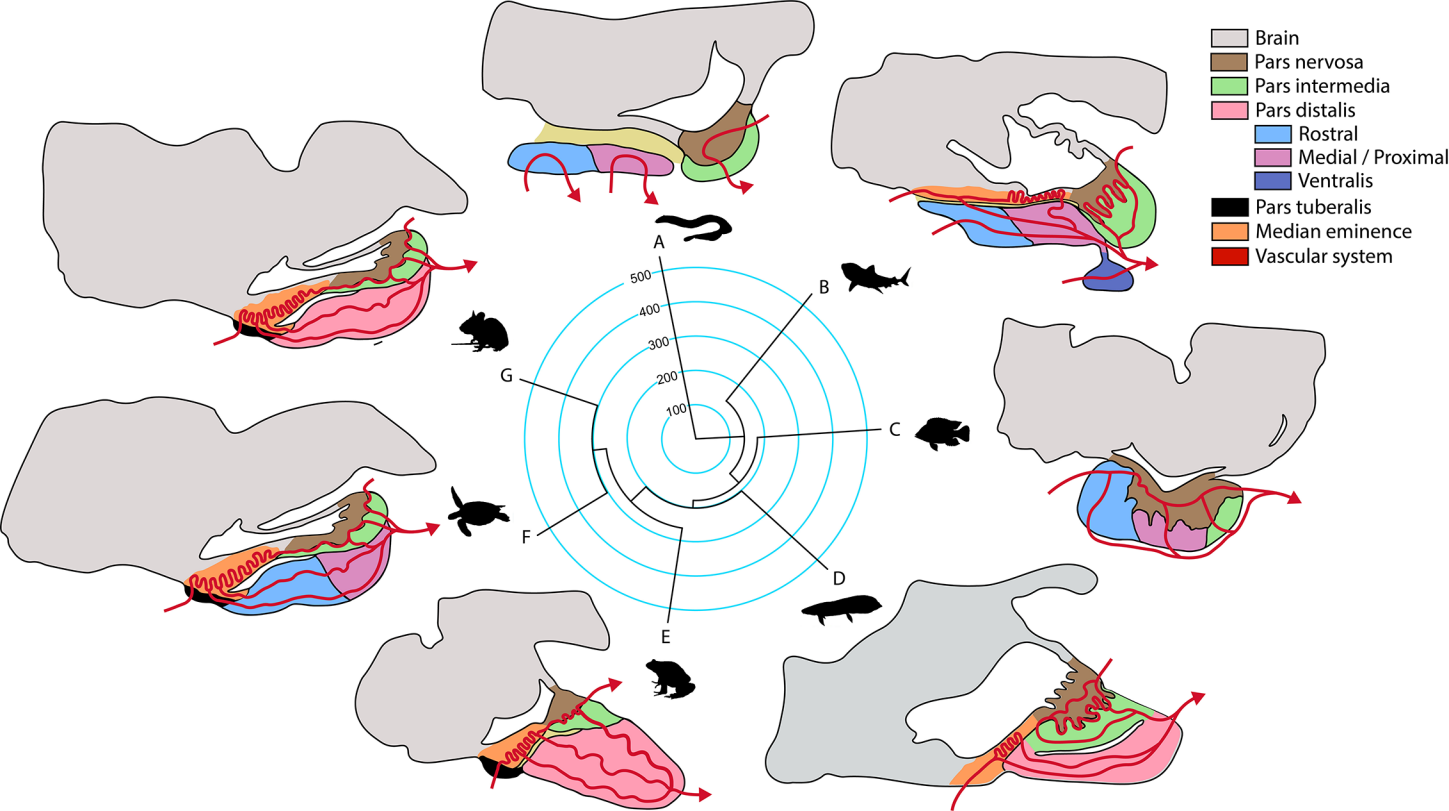
Phylogeny of the principal groups of vertebrates and anatomical pituitary organization (anterior-caudal-dorso-ventral orientation). Terminal branches of the phylogenetic tree show a representative organism of each clade (**A**, jawless fish; **B**, cartilaginous fish; **C**, ray-finned fish; **D**, lungfish; **E**, amphibian; **F**, reptile that includes birds; **G**, mammal) and their sagittal representation of the main structures in the pituitary gland according to (23). Median divergence times from the TimeTree database were used to construct the tree, numbers in the phylogeny are million years.

**Figure 2:**
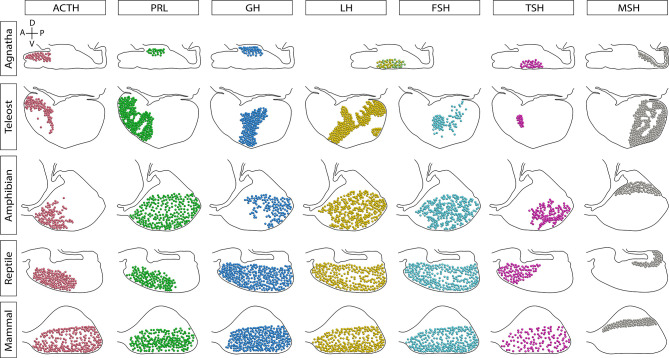
Sagittal representation of the endocrine cells distribution in the pituitary gland of vertebrates. The distribution of cell types has been modified over the evolution of vertebrates. In agnathans and teleosts, here represented by the sea lamprey and Nile tilapia, respectively, cells are markedly regionalized. Amphibians and reptiles present a regionalized distribution of some cell types, but others are scattered through the pars distalis. In contrast, the mammalian secreting cells are widely scattered throughout the lobe. In lampreys there is only one cell type producing glycoprotein hormone (GPH, a gonadotropin homologue) and thyrostimulin (TSN, a TSH homologue). Additionally, teleost fish possess a unique cell type that produces somatolactin, the somato-lactotroph, not shown. A, anterior or rostral; P, posterior or caudal; D, dorsal; V, ventral.

Over the course of vertebrate evolution, the general morphology of the pituitary gland has remained constant, with three main lobes whose function depends on the integration of signals originating from hypothalamic nuclei and from the systemic circulation (23). While a general anatomic plan is observed in the pituitary gland of all vertebrates (**Figure 1**), the specific arrangement of the endocrine cells (**Figure 2**), as well as the way by which their communication with the brain is executed, show great variation across taxa.

**Figure 3:**
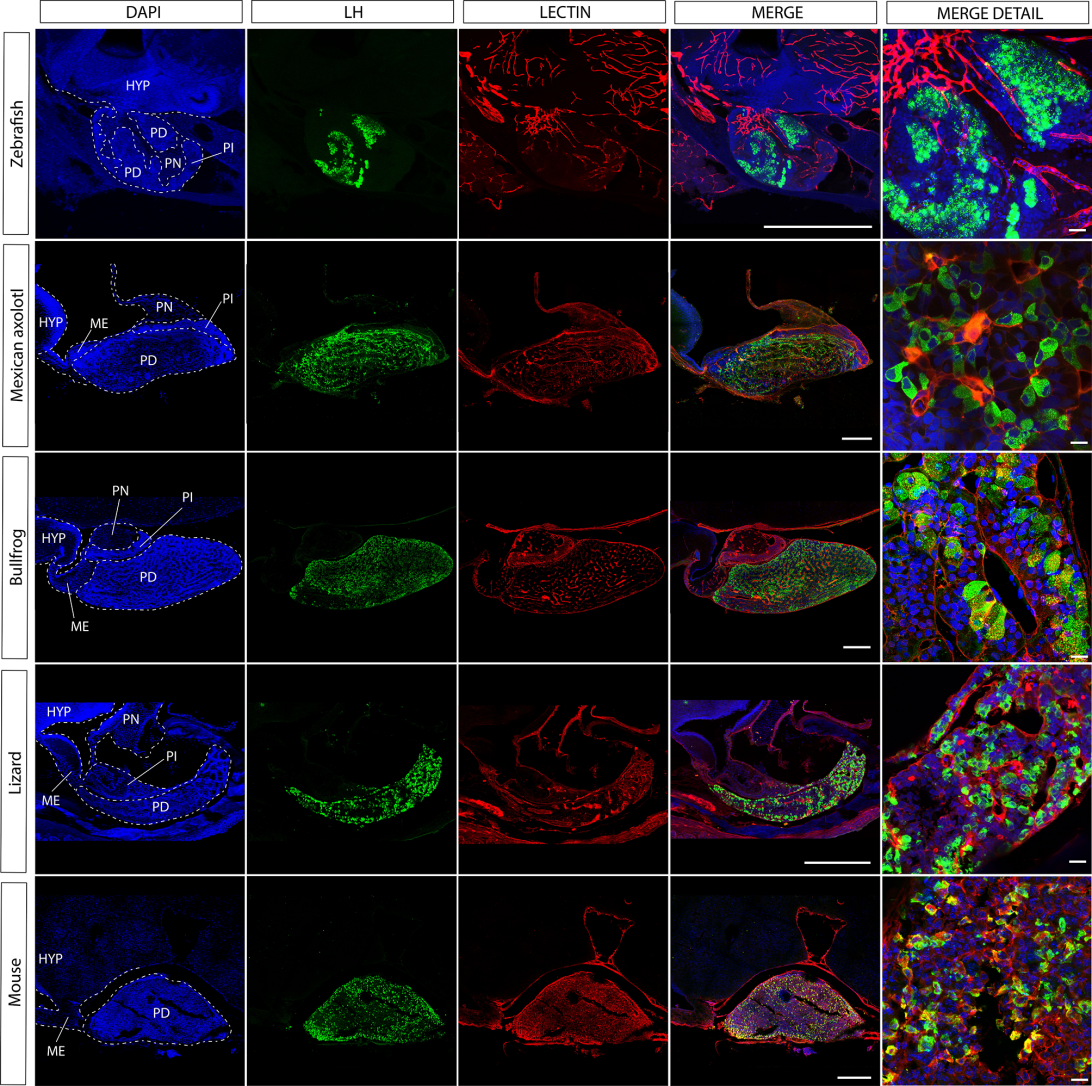
The gonadotroph network and the relation with the hypophyseal portal system in vertebrates. Midsagittal view of the pituitary gland in relation to the hypothalamus (anterior at the left, dorsal to the top) using confocal microscopy. Shown are immunostainings for LHβ (green) in five different species of vertebrates (zebra fish, *Danio rerio*; axolotl, *Ambystoma mexicanum*; bullfrog, *Lithobates catesbeianus*; lizard, *Sceloporus aeneus*; mouse, *Mus musculus*). In contrast to tetrapods, where LH-containing gonadotrophs are well distributed in the pars distalis, in teleost these cells are confined to the rostral pars distalis. The merge detail column is a power magnification of the precedent, showing that gonadotrophs establish cell-cell contact to form an interdigitated network and reveal that this pattern of cell organization is extended to all vertebrates. Note that the vascular network pervades the pars distalis and gonadotrophs form process to the capillaries (red, lectin-rhodamine), with the exception of zebrafish. DAPI counterstain is shown in blue. Dashed lines mark the three main regions of the pituitary. HYP, hypothalamus; PD, pars distalis; PI, pars intermedia; PN, pars nervosa; ME, median eminence. Scale bars represent 400 μm in whole-gland views and 20 μm for merge detail.

Teleost pituitaries in particular exhibit an important deviation from the masterplan of the tetrapod pituitary in respect to the anatomy of the hypothalamus-pituitary interface. Studies of fish pituitaries found that in ancient forms such as cartilaginous fish, sturgeons, and gars the hypothalamic fibers terminate and release their content onto the basal membrane separating the neurohypophysis from the adenohypophysis. The secreted hypothalamic factors then diffuse through the connective tissue and are uptaken by the pituitary vascular system that delivers them to their pituitary target cells (24). This neuro-vascular mode of delivery was perpetuated in the tetrapod evolution and is the dominant mode by which the hypothalamus exerts its effect on pituitary cells in all known tetrapods (24) (see **Figure 1**). However, studies of teleost pituitaries revealed a unique characteristic that was not found in any other vertebrate: hypothalamic fibers crossed the basement membrane that separates the neuronal tract from the adenohypophysis and formed terminals adjacent to endocrine cells (see teleost fish in **Figure 3**) (25–31). This unique characteristic drew so much attention, that a direct, neuroglandular mode of regulation was considered the main mode operating in teleosts.

The development of transgenic fish with labeling of three components of the HP axis (GnRH3 cells, gonadotropes and blood vessels) enabled researchers to re-visit the existing dogma. Upon the examination of the GnRH tracts in pituitaries labeled for gonadotropes it became clear that the direct neuroglandular mode of regulation cannot be considered as the dominant route of gonadotrope regulation. This is because not all gonadotropes are contacted by GnRH terminals and because the vast majority of these terminals are not in the vicinity of the gonadotropes (32). By using transgenic fish with labeled blood vessels it became apparent that the GnRH terminals are in much closer association with the pituitary afferent vessels than with the gonadotropes (see the teleost fish in **Figures 3** and **5**). This organization strongly suggests that, at least in zebrafish, GnRH signals reach their pituitary targets through the blood or through diffusion rather than *via* direct neuro-glandular contacts. A dominant neuro-vascular interaction is also observed in other species and other hypothalamus-pituitary axes (31–36).

The distinction between a direct, neuroglandular mode of regulation *versus* an indirect, neuro-hemal mode of hypothalamic factor delivery has important consequences over the functional physiology of the hypothalamic pituitary axis. A direct, neuroglandular mode of communication would require considerably smaller concentrations of hypothalamic factors to be secreted since the secretagogues will be delivered directly to the target cell. Such a direct communication mode may also affect the timescale of the regulation as a very temporally precise signal could be delivered to the target cells resembling the timescales of communication between neurons. Moreover, if the anatomical interactions between terminals and cells are very precise, it would make the variety of hypophysiotropic signals largely redundant as the specificity of the regulation could be achieved by controlling the type of neuron contacting a specific pituitary cell type rather than by different secretagogues. To date, there is no evidence to suggest that teleost pituitaries differ in any of these aspects from the more widely studied mammalian pituitary.

While it is admittedly difficult to evaluate the functional contribution of the different delivery modes, at least from a quantitative point of view, the direct, synaptic-like contacts between terminals and their pituitary targets seem to play a less important role than previously thought in the regulation of pituitary secretion. Instead, diffusion from terminals through the pituitary tissue to the target cells (either passive, or, like in tetrapods, assisted by local blood flow), appears to be the dominant way by which pituitary endocrine cells receive their hypothalamic signals. Therefore, the anterior neurohypophysis of teleosts may act as a functional median eminence that is embedded into the pituitary in teleosts. The evolution of the hypothalamo-pituitary axis has recently been thoroughly reviewed by Trudeau and Somoza (37) and interested readers are encouraged to refer to this excellent review for more detail.

In the next section we briefly cover the principal mechanisms involved in the maintenance of homotypic and heterotypic cellular networks in the pituitary, by means of cell-cell contacts or paracrine communication. Those mechanisms are present in all vertebrates but their contribution may vary depending on the communication with the hypothalamus, vascular arrangement of the pars distalis, distribution of cell types in the gland (regionalized or scattered), and formation of heterotypic networks (see **Figures 1** and **2**).

## Modes of Communication

### Gap Junctions

Gap-junctions (GJ) are transmembrane channels that allow the free cell-to-cell exchange of cytoplasmic molecules of <1,000 daltons molecular weight. The intercellular communication through GJ is an important source in the transport of cytoplasmic constituents (including water and ions), metabolic substrates (such as sugars, amino acids, nucleotides), and second messengers (mainly calcium, IP_3_, and cAMP) between cells. In the pituitary gland, gap junctions are an important mode of communications in all studied vertebrates. For example, gap junctions have been shown to couple fish pituitary cells (38, 39). In the rat, cells show a high level of intercellular coupling through gap junctions, where lucifer yellow diffusion was found up to 300-microns away from its site of injection (40). In addition, the transport between cells was primarily in lactotrophs, somatotrophs and folliculostellate cells (FS cells). Only a few LH, TSH, and ACTH cells were labeled (40). In all vertebrates gap junctions are considered a critical component in the coupling of pituitary cells and allow a coherent transmission of signals and the formation of cell networks (2, 19, 22, 41–48).

In terms of cell activity, Guérineau and collaborators have demonstrated the fundamental role of gap junctions in the synchronization of electrical activity (49). By multicellular measurement of spontaneous intracellular calcium mobilization in tissue preparations, the authors found the presence of clusters of pituitary cells that were in close proximity to each other and rhythmically coactive. As small molecules (Lucifer yellow, 457 Da), but not large molecules (Texas Red, 3 000 Da) spread between the co-active cells, in combination with the observation that a gap-junction blocker reduces the spread of synchronization, it was concluded that coordination between neighboring secretory cells is mediated by gap junctions. Of particular importance, the connected cells were principally of the same phenotype but not all the adjoining cells from the same phenotype were coupled. This fact indicated that the global response of secretory cells during hypothalamic stimulation involves, as we shall see, other mechanisms of cell communication that are not mutually exclusive.

Changes in gap junction expression patterns have been linked to plasticity in the pituitary gland. Modifications in connexins 43 and 26 expression (subunits of gap junctions), have been reported in rodents, following physiological challenges and aging (50–54). For example, GJ expression is modified according to the stage of the estrous cycle, probably reflecting the plasticity of cell connectivity during the generation of hormone pulses. Indeed, other authors have reported that estrogens play an important role in their regulation, since the absence of testosterone and estradiol downregulate the number of gap junctions by a quarter or more compared to intact rats (46, 55–57). Other experiments show that factors such as hydrocortisone, have a suppressive effect on the GJ formation between FS cells and between endocrine pituitary cells (58).

### Folliculostellate Cells

A population of glial-like cells, the FS cell network, is present in the pituitary gland of all major taxa of living vertebrates, and in mammals these non-endocrine cells comprise about 5–10% of the anterior pituitary cell mass (39, 59–62). These agranular cells have a characteristic stellate shape with long cytoplasmic processes, and arranged as a 3D anatomical network that extends over the whole gland in mammals (48, 53), aves (62), amphibians (63), and in fish (39). FS cells respond to a large variety of external and internal stimuli and produce growth factors and cytokines, such as fibroblast growth factor (FGF)-2, vascular endothelial growth factor (VEGF)-A, follistatin, and IL-6, all of which potentially have paracrine effects and regulate the activity of neighboring hormone-secreting pituitary cells (64).

The FS cell network has been suggested to wire the endocrine cells of the pituitary by relaying relevant signals throughout the gland. Secretory cells in the pituitary are intertwined through the vasculature and the FS network. The wave propagation of ions as Ca^2+^, and small molecules as IP_3_ or cAMP, through GJ within the FS cell network, allow synchronized excitability within the anterior pituitary gland (48, 65). Although, the distance and velocity in which the information can propagate to parenchymal cells has not been investigated yet, there is evidence supporting that the anterior pituitary possesses an intrinsic system of communication, that allows long-distance transmission of information within its parenchyma (66, 67). FS cells may have a central role in this process due to their gap-junction mediated network properties, and the expression of a large variety of signaling molecules. Hence, distant endocrine cells might receive coordinated information from both systemic molecules through the vasculature and paracrine signals primarily coming from the FS-cell network (48).

In mammals, it has been reported that FS cells can generate different responses in groups of endocrine cells surrounded by their cytoplasmic processes, thus forming functional clusters (66–68). Interestingly, it has been observed that GnRH-neuron projections converge not only at the blood capillaries, they are also adjacent to FS cells in the pars tuberalis (68, 69). Still, gap junctions between the GnRH-neuron terminals and FS cells, as well as GnRH-induced calcium response in FS cells have been recognized (69–71). In this sense, in addition to the vascular system, cell signaling in the FS network generated by hypothalamic stimuli, may be a route to propagate signals throughout the pituitary (66–69, 72, 73). In this respect, the unique anatomy of the teleost pituitary may provide interesting insights, since stellate cells have been reported to form dense networks around the neurohypophysis that penetrates deep into the parenchyma of the adenohypophysis (39). As a hypothesis to be proved, some endocrine cells may receive the signals only from the pituitary portal system and others could additionally integrate signals propagated by the FS-cell network, according to their distribution in the pituitary (70) (see **Figure 2**).

### Paracrine Communication

Paracrinicity is the process of short-distance communication between cells by substances released within the same tissue. The substance reaches its target by diffusion in the extracellular space or by direct contact formation (juxtacrine factors). More than 100 compounds in the pituitary have been identified as paracrine or autocrine factors (64). They include the neurotransmitters, growth factors, cytokines tissue factors such as annexin-1 and follistatin, hormones, ATP, NO, among others (64). The majority of paracrine or juxtacrine molecules, act through G-protein coupled receptors, with inhibitory (Gi) or excitatory (Gs) action, activating in most cases voltage gated channels activity and intracellular [Ca^2+^], as well as a whole host of other receptor types capable of affecting down-stream kinase and protein phosphorylation status (74). Paracrine signaling is a slow diffusion process, which occurs over seconds to minutes. This process of molecular diffusion is one of the main scenarios for possible organization and communication presumably sufficient to account for coordinated endocrine cell population activity and surge hormone output (74). However, this diffusion process seems to lack the directionality that would allow the formation of homo- or heterotypical networks, unless evidence is found to show that it could be a process directed through, for example, the interaction of molecules with the extracellular matrix. A more spatially precise intercellular signaling may be accomplished in the form of juxtacrine factors, as annexin 1 (formerly lipocortin-1), a phospholipid-binding protein which is predominantly expressed by the FS network (74).

In mammals, an extended review by Denef summarizes the paracrine interactions in the pituitary gland (64). The first evidence of the possible paracrine relationship between endocrine cells was established from gonadotrophs and lactotrophs, where the stimulation of gonadotrophs secretion with gonadotropin-releasing hormone (GnRH) triggers prolactin secretion (PRL) (75). More complex relationships were established between gonadotrophs and somatotrophs, in which it was observed that in aggregates of these two cell types, the GnRH stimulation produce an initial inhibition of GH release, whereas after GnRH stimulation, a rapid rebound secretion of GH was observed, suggesting that GnRH had both inhibitory and stimulatory actions on GH release (76). Gonadotrophs have a large number of secreted peptides who are potential candidates for a paracrine action. Some of these peptides modulate corticotroph activity when added exogenously to pituitary cell preparations (64). An inverse relation between lactotrophs and gonadotrophs was also established by the inhibition of LH secretion in hyperprolactinemia condition (77).

Other possible paracrine interactions imply coordination between different pituitary endocrine cells. For instance, the hypothalamic-pituitary-adrenal axis that is activated during stress. In this condition, there is a need to increase the metabolic rate in order to maintain body temperature, and this process is regulated by the hypothalamic-pituitary-thyroid axis (78). Further example of crosstalk with the HPT axis is during pregnancy and lactation, in which energy consumption has to be adapted. The mechanisms involved in the regulation of these homeostatic changes are still largely unknown but paracrine interactions are likely to be involved in the heterotypic network communication (64).

In fish, paracrine interactions between pituitary cells have been shown to comprise an important regulatory layer of pituitary cell function and probably operate at a slower timescale than gap-junction mediated transmission. Perhaps one of the most interesting aspects of this communication mode in fish is the interplay between the growth and reproductive axis through the paracrine effects of LH on GH cells. Studies in grass carp have shown that both GH and LH have a stimulatory effect on GH expression and release whereas GH has an inhibitory effect on LH release (79). Cross communication between the growth and reproductive axes in fish is also achieved *via* the activin/inhibin/follistatin pathway as these genes are expressed in the pituitary and have known effects on gonadotropin expression (80–83) and the GH-LH interaction (84). The expression and secretion of somatolactin (SL), a member of the GH/prolactin family, has been shown to be autoregulated by the two SL isoforms (85, 86) as well as by kisspeptin (87), a hypothalamic hormone that in fish is also produced in the pituitary gland (88). Other proposed autocrine and paracrine effects between different axes in the fish pituitary have been proposed such as the effect of NKB produced in gonadotropes (89) on lactotrophs and somato-lactotrophs (90). Secretoneurin-a, a potent stimulator of gonadotropes in goldfish is produced in lactotrophs of the rostral pars distalis and may link the two axes *via* paracrine effects on the gonadotropes of the proximal pars distalis (91). Recent findings in long-term culture adult medaka female pituitary, showed that, Fsh cells which do not possess Gnrh receptors start to express them, allowing Fsh cells to produce Lh. These observations suggest that *in vivo*, a paracrine signal inhibits Gnrh receptor expression in Fsh cells and maintains their mono-hormonal identity (21, 35, 92).

It is also possible that paracrine factors not just mediate homotypic and heterotypic network formation, but also could have a priming effect on axis function because in PRL cells, the increased organization associated with lactation persists for months after weaning and leads to enhanced function (93). Despite a large body of evidence, we are still far from understanding the full effect of paracrinicity on network formation, because it is technically difficult to isolate this process from others such as communication between GJ or electrical conduction in FS cells.

## Functional Pituitary Networks in Vertebrates

### Fish

One of the unique features of the fish pituitary is a high level of regionalization in the distribution of endocrine cells [(37, 94) and **Figures 2** and **3**]. This compartmentalization of the cells results in homotypic clusters of cells that favor direct cell-cell interactions and the formation of homotypic cell networks. Yet despite this unique topology, the study of cell networks in fish pituitaries has lagged behind that of mammals. The first evidence for direct cell-cell communication in fish pituitaries was the discovery of electrotonic cell coupling in the tilapia pituitary (38). Using double-patch electrophysiology on live pituitary slices, this groundbreaking work has shown for the first time the existence of gap-junction mediated communication between teleost pituitary cells. However, at the time, the type of coupled cells was unknown. Since then, many of the new insights regarding pituitary networks in fish were drawn from three model species: the zebrafish (*Danio rerio*), the Japanese medaka (*Oryzias latipes*), and Nile tilapia (*Oreochromis niloticus*). In these species, the generation of transgenic fish with labeled pituitary cells (35, 95–98) was an important turning point as it provided the opportunity to study topological cells networks in 3D and opened the way to functional studies of cell-cell interactions. Interestingly, in all three species, the main study of pituitary networks has concentrated on the gonadotrope cells of the anterior pituitary, leaving future studies to focus on other cell types. One of the reasons for this specific focus on gonadotropes stems from the fact that in fish, as opposed to tetrapods, LH and FSH are secreted from discreet cell populations rather than from a single cell type. This feature makes teleosts a particularly attractive model to study the differential regulation of LH and FSH secretion.

Studies in these three teleost species confirmed the existence of functional gonadotrope networks in fish. In tilapia and zebrafish, we have shown that LH cells are connected by gap junctions (22). In medaka, Karigo and co-authors used calcium imaging to show that in the intact gland LH cells exhibit a high level of synchronization in their activity (98) although the mode by which the cells communicate was not investigated. More recently, working with pituitary slices from medaka, Hodne *et al*. have used precise calcium uncaging in individual LH cells to show that the calcium signal can propagate to neighboring cells in a both homo-and heterotypic manner, leading to the conclusion that LH cells act as a relay for GnRH signals to FSH cells (35). The importance of the functional coupling has been revealed by its effect on hormone secretion from tilapia pituitaries. Application of gap junction blockers decreased GnRH-induced LH release from perfused pituitary fragments seven-fold (22), underscoring the role of the network in mounting an efficient response to hypothalamic stimuli. Interestingly, while tilapia LH cell output is highly attenuated by application of gap-junction blockers, FSH release remains largely unaffected. In medaka, calcium activity of FSH cells also seems to be less synched compared to their LH counterparts suggesting that the lower level of coupling between FSH cells may be a common feature of fish FSH gonadotropes. The notion that FSH cells act individually while LH cells mount coordinated responses is also evident in their secretion patterns since the induction of gonadotropin secretion *in vivo* causes a considerably stronger fold increase over basal levels in LH compared to FSH (89, 99–101).

Long extensions have been described in fish gonadotropes both *in situ* and in culture (32, 95, 102, 103). It is difficult to postulate as to the function of these processes but two plausible options are that these appendages are used for cell-cell communication or as a bridge between the cells and the vasculature. One line of evidence from cultured medaka cells (102) suggests that these processes do not relay signals between cells but are instead used for cell motility which serve to bring the cells together thus promoting cell-cell contacts and the formation of a network. It is hard to evaluate whether the processes serve the same role *in vivo* as in the adult medaka pituitary the cells are highly clustered yet the projections are still observed (102). Findings from the zebrafish model suggest that these processes are more likely to operate as extensions of the cells toward the vasculature than for intercellular communication. In the developing zebrafish, FSH cell extensions are clearly oriented in one direction: toward the primary plexus of vessels entering the pituitary (32). The aforementioned cytoplasmic processes are all directed toward the afferent vessels and terminate on their endothelium. This finding is even less surprising when considering the fact that not only metabolites are acquired from the blood, but also GnRH signals. At this early developmental stage no contact between gonadotrope and GnRH terminal exists. However GnRH fibers do reach the primary plexus, thus their output is carried through the blood to reach the developing gonadotropes (32). In addition to receiving blood-borne signals through the processes, these projections can also be used to deposit hormones to the circulation. In fact, medaka LH cell projections have been shown to develop large varicosities around the process-vessel junctions (102). It therefore seems more likely that the gonadotrope extensions are used to access the circulation than to mediate cell communication, though further investigations into this aspect are required.

While the study of pituitary cell networks in fish is still in its early stages, it is clear today that these networks developed early during vertebrate evolution and their function was conserved in higher vertebrates. In order to fully appreciate the role and function of pituitary networks in fish, future studies will have to extend the focus onto other pituitary cell types and probe the plasticity of these networks in response to physiological challenges that require the modulation of pituitary output.

### Amphibians

#### Metamorphosis

The process of metamorphosis in vertebrates is well represented by the post-embryonic morphological remodeling in amphibians, where changes in physiology and behavior also occur. During metamorphosis, the neuroendocrine system plays a major role orchestrating the modifications at cellular and molecular levels in tissues by thyroid hormones (104–106). Particularly, a peak of thyroid hormone secretion marks the beginning of the process and the thyrotrophs, from the hypothalamus-pituitary-thyroid axis, exert a direct action over the production of this peak (107). As thyroid hormone continues to stimulate tissue remodeling, a continuous activity of thyrotrophs in the pituitary is also needed to maintain the secretion levels of the former (108, 109). Thus, thyrotrophs must shift plastically the secretion pattern by remodeling their structure and their relation with other cellular types within the pituitary and feedback loops in the axis (108, 110).

It is noteworthy that TSH secretion during amphibian metamorphosis is stimulated by CRF, the hypothalamic factor from the stress axis and not by TRH (111, 112). It is now understood that in amphibians, TRH activates TSH secretion in thyrotrophs of metamorphosed individuals but not in larval ones as reported in the bullfrog, reflecting that the thyrotroph plasticity is modified ontogenetically (113, 114). This phenomenon implies that thyrotrophs express receptors for TRH and/or that corticotrophs may regulate TSH secretion through paracrine communication (110, 115, 116). To date, the physiological and structural relationship between corticotrophs and thyrotrophs in the process of metamorphosis and whether a heterotypic network is formed remains to be elucidated, but it is possible to speculate about their intimate relationship, based on the shared main hypothalamic factor that regulates these cells types and also that the stress axis participates actively during the metamorphosis and its developmental plasticity (110, 117–120). Furthermore, cell plasticity in other pituitary cell types is also observed during metamorphosis. For example, when the salamander *Hynobius retardatus* was arrested prior to metamorphosis, an increase in the number of TSH-secreting cells was observed as well as an increase in bihormonal phenotypes such as TSH-PRL and TSH-GH, presumably involving transdifferentiation (121, 122).

The hypothalamus-pituitary-thyroid axis is also intertwined with the reproductive axis, where amphibian TSH in combination with gonadotropins has been demonstrated to trigger gonadal maturation (122, 123). Metamorphosis may modify gonadal maturation under environmental influence as demonstrated in urodels with neoteny, the heterochronic process where the larval phenotype is retained but organisms are sexually mature (124). Studies comparing the modification in the pituitary networks before and after metamorphosis in pedomorphic species (e.g., *Ambystoma mexicanum*; see **Figure 3**) with those with direct development (e.g., the bullfrog; see **Figure 3**) may reveal some of the molecular mechanisms involved in network plasticity, but also about its interaction with the hypothalamus (125–127).

#### MSH Cells

Melanotrope cells are essential for amphibian camouflage *via* secretion of alpha-melanophore-stimulating hormones (α-MSH), a hormone derived from the POMC protein (128, 129). The melanotroph cells are restricted to the pars intermedia; in contrast to other secretory cells in anterior pituitary that are heterogeneously distributed and where heterotypic contacts are formed. The cluster organization of melanotrophs, must permit a fine coordination in response and α-MSH secretion, and probably involves gap junctions since expression of connexins (Cx43) has been reported (130, 131). The vascular system is a second candidate for melanotrope cells organization and remodeling. In fact, in normal conditions, the pars intermedia is poorly vascularized compared to the adenohypophysis (see **Figure 3**), the capillary networks are present at the borders between the neural lobe and intermediate lobe (**Figure 1**). However, in transgenic frogs (*Xenopus laevis*) that overexpress VEGF-A in the pars intermedia, there is a complex formation of a vascular network (132). VEGF-A, the vascular endothelial growth factor, is involved in the formation of the vascular system during the development of the hypothalamus-pituitary system, and in the transgenic *Xenopus*, the increase of the vascular network has several consequences in the pars intermedia. First, these new capillaries are distributed around the lobules formed by melanotrophs. Second, the intermediate lobe is up to 3.5-fold greater than in control frogs as a consequence of melanotroph hypertrophy and hyperplasia, but the pars distalis is not reorganized. The implication of these results pointed to the active role of the vascular network in pituitary function and homeostasis. Further studies will be required to understand the effect of the vasculature in the pars distalis where cell networks are differentially disposed to the capillaries (see **Figure 3**). Specific modifications in the vascular system of animal models, as the example offered here, may also reveal how endocrine diseases emerge as vascular problems (2, 133).

### Reptiles and Birds

The establishment of cellular networks in the reptilian pituitary has not been studied directly, but the available literature indicates that homotypic and heterotypic networks may exist. For example, close contact between cells from the same lineage (including thyrotrophs), cluster formation, and cytoplasmic extensions have been reported by immunohistochemical studies (134–136). Due to their phylogenetic position between amphibians and mammals, reptiles gain relevance in the study of evolutionary transitions in the pituitary structure and function. Of particular importance may be the investigation of the chronological appearance of pituitary cell types, a process where ontogeny probably does not recapitulate phylogeny. Based on the work of Yamaki and collaborators, it has been demonstrated that the sequential development of endocrine cells in the mammalian pituitary varies largely in other vertebrates without a clear pattern, even close species show diversity in pituitary developmental programming (137). As an example, the establishment of the gonadotroph network upon the corticotroph scaffold, as described in mammals (138) may not be the case in turtles or fish, where corticotroph cell distribution is principally restricted to the rostral pars distalis, although gonadotrophs are widely distributed in the lobe (**Figures 2** and **3**). Comparative cell lineage in combination with other methods, such as lineage tracing, single RNA sequencing, and functional studies will reveal the mechanisms underlying the structure of pituitary networks, the heterochronic appearance in vertebrate development and their plasticity.

### Mammals

Of all vertebrates, pituitary networks in mammals, and particularly in mice, have been studied in the most comprehensive manner and their existence has been demonstrated in lactotrophs, gonadotrophs, somatotrophs, corticotrophs, and folliculostellate cells (2, 16, 19, 20, 48). The modes by which mammalian pituitary cells communicate have been described earlier. Unlike in other vertebrate classes, works in mammals have focused not only on the existence of pituitary cell networks, but also on the way by which these networks modulate their function to meet the changing demands of the animal.

#### Lactotrophs

The process of lactation requires a big amount of prolactin production and secretion from pituitary by lactotrophs. Milk production requires a 10–50-fold increase in prolactin secretion and involves a marked decrease of dopamine production by neurons in the hypothalamus (18, 139). Dopamine normally inhibits prolactin secretion through dopamine receptors coupled to inhibitory G-proteins that block the voltage gated calcium L-channel and change the membrane potential, but also through a short-loop feedback mediated by prolactin itself. However, during lactation DA-producing neurons switch from inhibiting prolactin secretion to promoting its release by secreting enkephalin (139). Although necessary, these hypothalamic changes do not account for the major alterations that support the increase in prolactin secretion (2).

In 2012, Hodson and collaborators reported the existence of physiological and morphological plasticity in the lactotroph network that reinforce the secretion activity of these cells and were not directly dependent on hypothalamic activity (19). First, from experiments of intracellular calcium measurements in lactotrophs, it was shown that during lactation, the network functional connectivity is more robust as both calcium coactivity between pairs of cells and correlation in calcium profiles increase. Remarkably, in virgin mice the network connectivity of lactotrophs is low and only few cells harbor the majority of these connections. In contrast, in lactotrophs from lactating mice there is an increase in the proportion of significantly correlated cells and new nodes emerge. This last characteristic of the network persists after weaning, even months after the lactation event is over. Second, increase in connectivity is not a direct result of the decrease in dopamine production, since in virgin mice treated with a dopamine-receptor antagonist, an increase in functional connectivity is not observed. Furthermore, the interruption or decrease in suckling stimulus during lactation significantly reduces this functional network at values to those observed in virgin mice. Third, the coordinated functional activity in the network is directly dependent on structural connectivity due to lactotroph hypertrophy and gap junctions. When a gap junction blocker was applied to the lactotroph network from a lactating female, the connectivity resembled the network of virgin mice. Finally, the plastic transition of this network remains assembled and is crucial in subsequent lactation events that are characterized by a prolactin secretion improvement (19). The effects of the lactotroph network reconfiguration on other networks, for example gonadotrophs and reproductive axis, and the heterotypic interactions remain open questions, but their physiological relevance is clear.

#### Gonadotrophs

Another process where the hypothalamic regulation of pituitary does not account for the shift in hormone release is offered by gonadotrophs and LH secretion throughout the estrous cycle. LH and FSH secretion are necessary for follicle maturation and ovulation in females but they are differentially secreted through the reproductive cycle (140). For example, in proestrus the pattern of LH pulse secretion turns into a surge mechanism that has been largely seen as a consequence of the GnRH surge, the main stimulating factor of gonadotrophs in the hypothalamus. However, GnRH stimulus *per se* is not the causal phenomena for the significant increase in responsiveness and secretion by gonadotrophs (140, 141).

*In vitro*, it has been reported that during diestrus, the gonadotroph response to GnRH is limited and just a small proportion of the population present intracellular calcium activity (142). High concentrations of GnRH (100 to 1,000 nM) are necessary in this state of the cycle to produce the whole-population response, but the amount of LH secretion does not increase significantly. On the other hand, in proestrus, low GnRH concentrations are sufficient to trigger a whole population response and, more importantly, the majority of gonadotrophs secrete a larger amount of LH. Despite the increase in gonadotroph responsiveness and LH secretion during proestrus that depend upon GnRH receptor expression, these processes also involve the gonadotroph network reconfiguration and its relation with vasculature (143).

From experiments *ex vivo*, where tissue interactions are preserved, the gonadotroph network reveals dynamic and plastic adaptation in proestrus through an increase in cell number (probably involving cell proliferation and transdifferentiation). Gonadotrophs also increase cell-cell contacts (involving cell motility), and the increase of protrusions toward other gonadotrophs and to the vascular systems (141, 143). Taken together, these cell modifications and changes in responsiveness to GnRH during the estrous cycle, account for the regulation in the rhythm and mode of LH secretion at different phases of the reproductive cycle. The plasticity in gonadotrope cells at the molecular, cellular and population levels and a comparison of shared mechanisms between fish and mammals has been recently reviewed and for a more in-depth description the reader can refer to (21, 144).

Plasticity in gonadotrophs is canalized in a context-dependent manner: inputs from the reproductive axis as well as from the environment are interpreted within the network to activate or attenuate the system output. For example, testosterone, estradiol, and GnRH have been reported to stimulate the LH secretion, cell proliferation, and GnRH receptor expression when present at certain concentrations and rhythmicity, but may desensitize the network and inhibit the LH production and secretion when present for prolonged periods of time or higher concentrations (21, 145).

**Figure 4:**
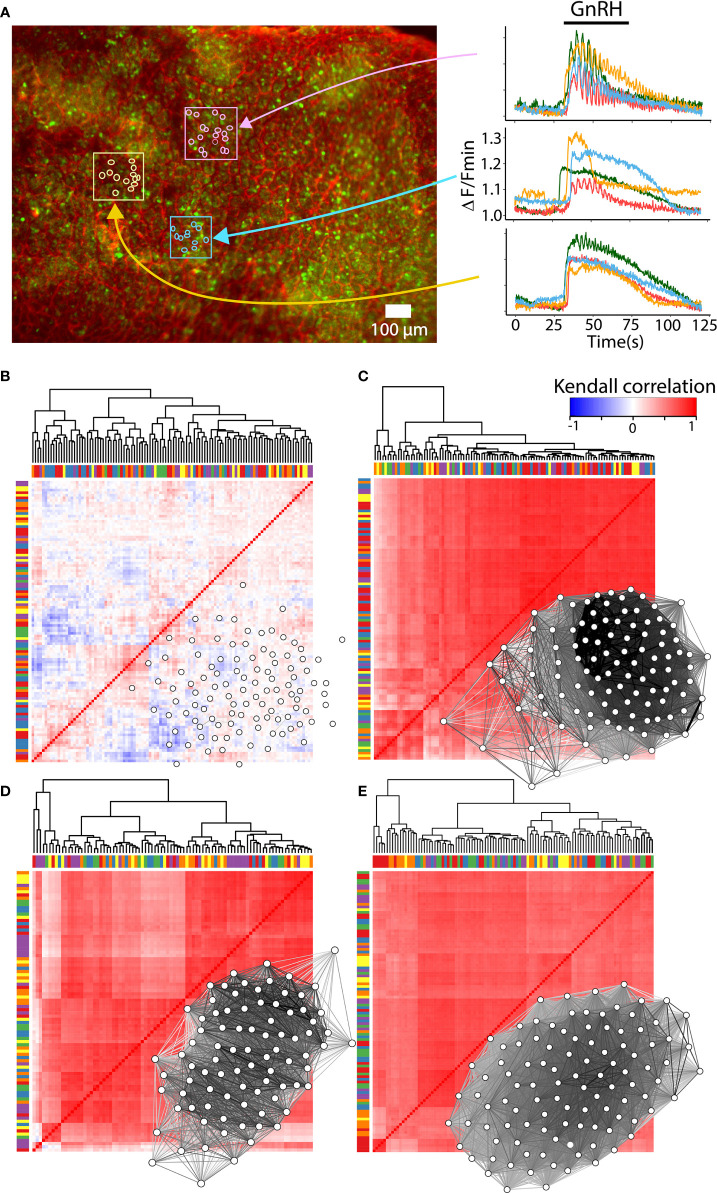
Reconfiguration of the gonadotroph network throughout the reproductive cycle. **(A)** Intracellular calcium recordings in *ex vivo* preparations of mouse pituitary loaded with the calcium sensor Fluo 4-AM (green) and rhodamine for vasculature (red). Monitoring of calcium activity was performed on the ventral side of the gland under an epifluorescence stereomicroscope and continuously perfused with Ringer’s solution [methods as in (146)]. Color traces show the intracellular calcium activity of individual gonadotrophs in response to 10mM GnRH, indicating clusters of gonadotrophs surrounded by vasculature with a similar (pink and yellow boxes), or heterogenous (blue box) calcium response. **(B)** Heatmap and network organization of basal calcium activity of correlated pairs of gonadotropes from a male mouse. Gonadotrophs stimulated with GnRH in male mouse **(C)**, female mouse in diestrus **(D)**, and proestrus **(E)** showed an increase in synchronized calcium activity compared to basal activity. Notice that the overall correlation values are similar between diestrus and proestrus, as well as their connectivity. Bars in the heatmaps indicate sorting of gonadotropes by correlation but not by spatial proximity, while groups of gonadotrophs surrounded by vasculature are represented with the same color in bars. Network maps were plotted using qgraph and significant pairs (edges) of correlated cells (nodes) are shown (P < 0.05).

Most factors involved in the network establishment are necessary but not sufficient for a complete explanation of the mechanisms involved in the gonadotroph function as a network. GnRH is an element that triggers the network response since the intrinsic activity of gonadotrophs is not particularly synchronized and is not associated with gonadotropin secretion (see **Figure 4**) (147). Furthermore, the amount of gonadotropin secretion is influenced by the balance of hormones in the reproductive axis and includes a reconfiguration in the network connectivity and this change is associated but is not a consequence of the GnRH stimulus (141, 143). Such connectivity is principally constructed through soma-soma contact and cytoplasmic extensions in which gap junction-mediated intercellular communication or cytosolic connection are involved. However, if the gonadotroph network is wired only by cell-cell contact, one could expect that cells in close proximity to each other, should present a similar pattern of response and form clusters where degrees of synchronization may be highest (19, 47). Interestingly, the analysis of clusters by correlation in the patterns of calcium mobilization shows that distant cells respond to GnRH similarly, but cells in contact or surrounded by capillaries may exhibit different responses, indicating that other mechanisms such as paracrine communication or connectivity with FS cells play an important role in the network function (64, 148, 149) (**Figure 4**). In addition, the functional and structural connection in the gonadotroph network is plastic and remodels throughout the estrous cycle, probably following the physiological demand during the proestrus of an LH surge (143). The variety of molecules and processes involved in the network formation, their relative contribution, as well as their degree of plasticity requires further investigation.

#### Heterotypic Interactions

The homotypic network organization of gonadotrophs and lactotrophs discussed before provide evidence for its physiological relevance and plasticity. GH-secreting cells are the third population in pituitary that are organized in a homotypic network. This network is reconfigured during puberty with a sexually dimorphic pattern, probably as a result of different and dynamic feedback exerted by gonadal steroids (8, 150, 151). To date, the homotypic GH-cell network is the most extensively studied population in the anterior pituitary. There is evidence demonstrating the intimate interaction of the somatotroph network with other cellular types forming exquisitely arranged heterotypic networks, and when these interactions are disrupted, there are important repercussions at the gland-level hormone secretion (150, 152–155). For example, the ablation of somatotrophs at different degrees, compromises the pituitary production of TSH, PRL, LH, and ACTH, resulting in a modified ultrastructural composition of the cells and pituitary hypoplasia (152, 156, 157). Importantly, GH-cell ablation also impairs cell-cell communication between this homotypic network and other cell types, instead of causing GH reduction. The mechanisms of buffering the complete loss of somatotrophs in this model suggests an overproduction of the lineage from progenitor cells and a reduction in the generation of other lineages (152, 156, 157). Other possibilities could reflect transdifferentiation from different cell types since lactotrophs have been reported to decrease in number in these conditions, and it may also reflect the plastic function of multiresponsive cells that have been observed to increase under several physiological challenges (158, 159). However, the mechanisms involved in this process remain to be explored.

Less understood is the ontogeny of the homotypic and heterotypic networks in the pituitary gland. The observation of the complex arrangement of networks leads to the question regarding the developmental origin of cellular interactions and two options are possible. First, it is possible that at the point of cell proliferation of one lineage, cells never lose the contacts (such as cytonemas) and form a continuum allowing high synchronicity of activity and identity prevalence of the homotypic networks, as observed in the first stages of spermatogenesis (160). Alternatively, the cells might appear isolated, establish a position in the gland, and after that, the lineages could acquire the connectivity observed in adulthood. The available literature revealed that the second option is observed during development in the corticotroph and gonadotroph networks (138). Corticotrophs and gonadotrophs first appear in the ventral side of the pituitary as small cells without cytoplasmic projections, and isolated from other cells of the same lineage. Then, corticotrophs reshape their morphology, penetrate into the adenohypophysis, and interconnect with other corticotrophs and with the vasculature through cytoplasmic projections (138). Gonadotrophs follow the same pattern and it is reasonable to speculate that the organization of other networks in the gland also appears just after terminal differentiation (16, 138).

The sequential differentiation of cell types in the pituitary also suggests that homotypic interactions establish first and the heterotypic networks appear later, although the lineages that are developmentally older have a repercussion over the newborn cells and lineages (156, 161). For example, LH-positive cells first appear in the ventral surface of the pituitary at embryonic day 17.5 when the homotypic network of corticotrophs is already established. LH cells then form a homotypic network using the corticotroph network as a scaffold. In fact, blocking cell differentiation of the POMC lineage leads to an increase in the number of LH-expressing gonadotrophs that are mainly located where corticotrophs should be distributed (138, 162).

One may ask whether the increase of the gonadotroph population after the interruption of corticotroph establishment during development is due to a physical or morphogenetic constraints exerced from corticotrophs to the other populations. The idea that corticotrophs and gonadotrophs probably arise from a common precursor, suggests that the extended gonadotrophs lineage in deficient POMC cell differentiation is more than expansion of the former in an “empty niche,” reflecting a complex feedback between networks. For example, the transcription factor Tpit, expressed exclusively by POMC cells, exerts a negative effect over SF1, the transcription factor associated with the establishment of the gonadotroph phenotype (162, 163). This and other molecular interactions (e.g., pit-1 blocking the expression of the gonadotroph-like phenotype in thyrotrophs throughout GATA-2), suggests that a molecular interplay exists between cell lineages and results in an equilibrium of cell populations (16, 138, 164). The dynamic equilibrium achieved during ontogeny could persist to adulthood and shift to different points of equilibrium in a context-dependent manner, with the possibility of return to previous states, revealing the substrate of a highly plastic gland with cell populations that are able to navigate toward alternative phenotypes [see for example Sox2-expressing cells in adult organisms (156, 159, 165, 166)].

To date, other heterotypic interactions between the five mammalian adenohypophyseal populations have not been investigated but there are reasons to speculate about their relation and interdependence. First, the interlocked cell arrangement of gonadotrophs and corticotrophs was uncovered recently only by 3D image analysis that is hidden under classical 2D microscopy analysis (138). In this sense, thyrotrophs, the less abundant secretory population, may reveal a 3D continuous distribution in the pituitary [but see (130)]. Second, under diverse physiological challenges, transdifferentiation between cell types occurs independently of their ontogenetic proximity. For these reasons, the proportion of multi-responsive and multi-hormonal cells are constantly fluctuating, and different phenotypes such as lacto-somatotrophs, somato-gonadotrophs, gonado-lactotrophs have been observed (144). Finally, as we have mentioned before in this work, paracrine communication may play an important role in heterotypic communication, although its full implications are not yet clear (64).

## Conclusions

**Figure 5:**
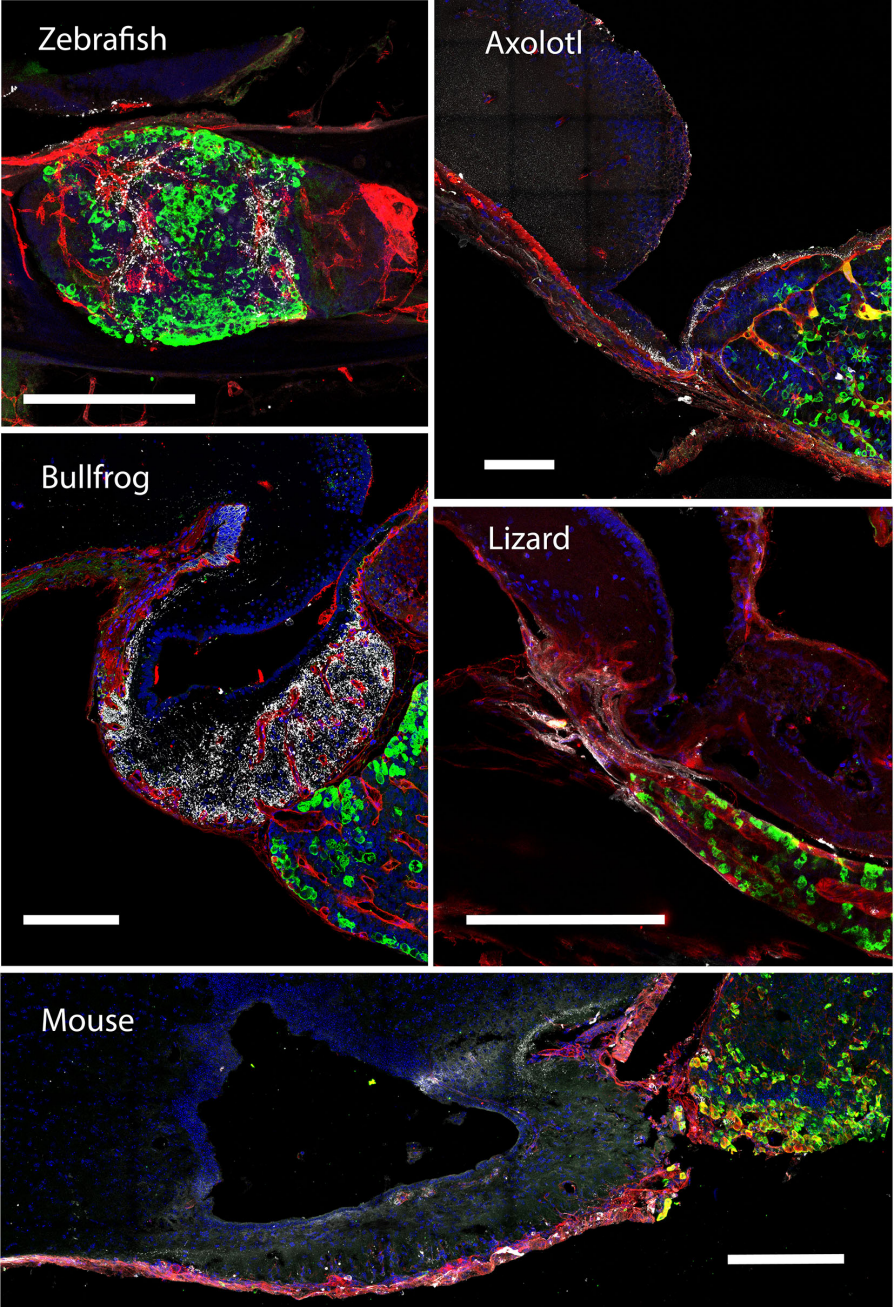
A multimodal communication between the hypothalamus and pituitary cell networks. Shown are sagittal sections (20 μm) of 4% paraformaldehyde fixed tissue, anterior at the left, dorsal to the top. Immunocytochemistry for gonadotrophs containing the LHβ subunit (green) and GnRH neurons (gray), species as in **Figure 3**. The distribution of gonadotrophs in the pars distalis, proximal to the hypothalamus, have remarkable cell-cell contacts in the tetrapod species, in teleost these cells are exclusively found in the rostral pars distalis forming a clear-cut cluster. The gonadotroph network arrangement is highly influenced by the vascular system (red; lectin-rhodamine), since these cells have cytoplasmic extensions to the capillaries. In tetrapods, the GnRH-producing neurons extend their projections to the median eminence where GnRH is secreted to the capillaries. In contrast, the GnRH neurons in teleost unfold throughout the pituitary and establish at the boundaries of the gonadotroph patch, following the vascular system. Note the close proximity between gonadotrophs and GnRH at the median eminence of tetrapods. DAPI is shown in blue for counterstain. Scale bars represent 200 μm.

Despite variations in the hypothalamus-pituitary communication and the organization of the cell populations within the gland, pituitary cell networks have persisted throughout evolution, revealing their pivotal role in the ability of the pituitary to mount effective responses to hypothalamic and systemic stimuli. Two major elements allow the formation of networks in the pituitary gland: cell-cell contacts and diffusible factors, both present in vertebrate species, although with different preponderance. For example, in fish, where cell types are present in clusters and hypothalamic innervation invades the pituitary gland (see **Figure 5**), the secreted factors by these neurons near the vasculature reach their pituitary targets by diffusion. However, as not all cells in a cluster (see gonadotrophs from **Figure 5**) are in close proximity to neuron terminals and capillaries, a synchronized response is also achieved by gap junctions. Furthermore, the relevance of the vascular systems may reside in the delivery of nutrient and oxygen supply as well transport of pituitary outputs to the blood circulation, meanwhile paracrine and FS cells could produce a balance between distinct secretory cells and neuroendocrine axes. In contrast, in mammals, where the endocrine cells are distributed along the gland and the hypothalamic neurons secrete to the vasculature in the median eminence, a smaller proportion of the network could receive hypothalamic factors by diffusion (**Figure 5**). It seems that homo and heterotypic communication allow coordinated propagation of signals between distant cell subpopulations, that may be enhanced through FS cells, paracrine factors and gap junction in small spaces (100 μm). Moreover, in other cell types that are less proximal to the median eminence (e.g., thyrotrophs), probably the hypothalamic signals are initially delivered through the vasculature and propagated by FS afterward. In summary, plasticity and evolution of the pituitary gland is the tinkering of existing parts combined in new ways rather than the creation of new parts.

In all vertebrate species, different physiological changes such as development, metamorphosis, reproductive cycles, stress, and metabolism require different pituitary outputs, not limited to large changes in the amount of secreted hormones, but also in timing and pulse frequency. Therefore, the gland and its interactions with the hypothalamus, organs, and tissues of the body are constantly being remodeled. The integration of all signals and the generation of hormone pulses, is the result of the plasticity of pituitary cell homotypic and heterotypic networks. Therefore, the interaction between these endocrine networks, are an important driver of the organism homeostasis.

The plasticity of hormone secretion in the pituitary gland has largely been seen as the result of changes in the hypothalamic input and pituitary-intrinsic modulations such as cell proliferation, cell size modifications, transdifferentiation, and multi-responsiveness. However, this review and other authors have contributed to the discussion of the elements that support the complexity of hypophysial plasticity. Several elements are involved to achieve this plasticity and have been discussed in detail (16, 20), these are: a) *gain of function*, by integration of stimuli from neuroendocrine axis and systemic information in the interaction of axes, as GH and PRL-networks, whose coordinated response to GHRH and TRH, respectively, is not seen when cells are dispersed; b) *plasticity for hormone secretion*, during changes in physiological demand, as seen in puberty when the GH axis is highly active and there is an increase in male somatotroph clustering associated with large amplitude of GH pulses; c) *experience-dependent response*, that is improved as a consequence of repeat demand, as mentioned in lactotrophs during the process of lactation; d) *network support of transcriptional synchronization*, when gene transcription follows a rhythmic pattern at the population level that is lost in dispersed cells, as it happens in lactotrophs; e) *redundancy through modularity*, although all the cells in a homotypic network are connected, there are subgroups of cells connected more strongly than other, this fact promotes certain independence of activity, and perturbations in a given region not necessarily affecting the whole network given the possibility of local homeostasis.

It is noteworthy that, despite their enormous functional importance, the study of pituitary networks is still in its infancy. In the last years there has been more literature focusing on the study of the pituitary as a system with functional networks. The term “network(s)” associated with the pituitary gland, as a main topic, first appeared in scientific literature in 1990, with the study of avian adenohypophysis and the effect of a serotonin precursor on thyrotrophs and the follicle-stellate network (167). In the next years, the concept of pituitary networks became popular and just in the last 10 years, 890 publications have been reported using the term network as a topic, according to The Web of Science Core Collection. Research of pituitary networks refers to different levels of interaction including protein-protein, protein-DNA, protein-metabolite, and cell homotypic and heterotypic networks (20). Accordingly, this young research field is highly productive and growing rapidly, showing the relevance of the cellular interactions when studying plasticity of the gland under diverse physiological demand.

So far research has focused mainly on the mammalian model of mice and, more recently, on fish. Research in both of these organisms has relied heavily on transgenic animal models that allow the identification, monitoring and manipulation of specific cell populations in live tissue as well as 3D imaging of the cell networks. Extending the study of functional pituitary networks to other vertebrate classes will be greatly assisted by the generation of transgenic animals in these taxa.

The development of genetically encoded calcium indicators (168) and *in vivo* imaging techniques (169) are expected to play an important role in advancing our understanding of the function and plasticity of pituitary cell networks in the coming years. These methods will allow researchers to extend the study of these networks from slices to freely moving and behaving animals and will therefore significantly increase the physiological relevance of our insights. Furthermore, single-cell transcriptomics (5, 170) and precise genome editing through viral delivery (171) will allow researchers to reveal the molecular machinery that drives the function and plasticity of pituitary cell networks, the relative role played by the elements that establish these networks and the relevance of the multimodal communication in the hypothalamus-pituitary system.

## Author Contributions

TF and MG: Conception of the line of work, structure of ideas, and writing. YS-A: Writing, immunocytochemistry, calcium recordings, and figures. All authors contributed to the article and approved the submitted version.

## Funding

Authors were supported by grants from CONACyT and the Agence Nationale de la Recherche (273513, Repro-Net) and PAPIIT (IN222613 and IN227416). YS-A was supported by CONACyT master funding.

## Conflict of Interest

The authors declare that the research was conducted in the absence of any commercial or financial relationships that could be construed as a potential conflict of interest.
